# Sicker at all ages? Two decades of changing disease histories and mortality in the United States

**DOI:** 10.1093/ije/dyag217

**Published:** 2026-10-10

**Authors:** Marcus Ebeling, Neil K Mehta, Leah Abrams, Mikko Myrskylä

**Affiliations:** Max Planck Institute for Demographic Research, Rostock, Germany; Institute of Environmental Medicine, Karolinska Institute, Stockholm, Sweden; Max Planck – University of Helsinki Center for Social Inequalities in Population Health, Rostock, Germany; Department of Epidemiology, University of Texas Medical Branch, Galveston (TX), United States; Department of Community Health, Tufts University, Medford (MA), United States; Max Planck Institute for Demographic Research, Rostock, Germany; Institute of Environmental Medicine, Karolinska Institute, Stockholm, Sweden; Max Planck – University of Helsinki Center for Social Inequalities in Population Health, Rostock, Germany; Helsinki Institute for Demography and Population Health, Helsinki, Finland

**Keywords:** disease history, prevalence, mortality, population composition, disparities

## Abstract

**Background:**

Stagnating cardiovascular disease mortality and rising drug- and suicide-related deaths are key drivers of stalled US mortality improvements. However, prior research has largely overlooked disease histories preceding death, despite increasing multimorbidity. We examined changes in disease-history profiles in the US population and assessed how shifts in profile composition and within-profile mortality shaped mortality trends.

**Methods:**

We analysed adults aged ≥30 years (*N* = 510 372) from the IPUMS-NHIS (National Health Interview Survey) 1997–2018 linked mortality files (follow-up through 2019). Latent class analysis on the pooled sample across all ages and years identified disease-history profiles based on self-reported cancer, diabetes, cardiovascular disease, mental health, internal diseases, obesity, and physical limitations. Logistic regression was used to estimate profile-specific 5-year mortality over time, and counterfactual decomposition was used to quantify the contributions of compositional change and within-profile mortality improvement to overall mortality trends.

**Results:**

Five disease-history profiles were identified. Prevalence and 5-year death probability ranged from disease-free (62.4%; men 3.7% [95% confidence interval (CI) 3.5–3.8], women 2.4% [95% CI 2.3–2.5]) to high-burden multimorbidity (5.9%; men 17.8% [95% CI 16.8–18.8], women 12.6% [95% CI 11.9–13.3]), with substantial gradients across intermediate profiles. Profile composition varied substantially by gender, education, and race/ethnicity. The disease-free population declined by nearly 1% annually, replaced primarily by cardiometabolic and high-burden multimorbidity profiles. Most profiles experienced stagnating or increasing mortality after the mid-2000s. The decomposition shows that within-profile mortality improvement was the dominant driver of declining mortality; compositional shifts, mostly driven by the declining disease-free share, partially offset these gains.

**Conclusion:**

Stagnating US mortality reflects both the growing prevalence of high-morbidity profiles and worsening or stagnating survival within them, highlighting the limited reach of prevention and disease-management efforts.

Key Messages

- Americans today enter midlife with more complex and burdensome disease histories than they did two decades ago.
- Disease-history profiles reveal how several unfolding health crises—rising cardiovascular disease, increasing obesity, and growing mental health and despair-related burdens—intersect within the US population.
- Stagnating US mortality reflects both the rising share of adults with high-morbidity disease histories and worsening survival within these groups, highlighting the limited reach of prevention and chronic-disease-management efforts.

## Background

There has been a crisis in US population health over the past 15 years, most often characterized by a slowdown in mortality improvements [1–4]. Research to date has revealed several explanations [5], including stagnation in cardiovascular disease (CVD) mortality, particularly among older populations, and increasing deaths from drug overdose and suicide, primarily among working-aged adults [6, 7]. While research on mortality trends has provided important insights into US population health, it has notable limitations. Most critically, mortality data reflect a snapshot of life’s endpoint, offering limited insights into the prolonged and complex disease histories that unfold throughout the life course [8]. This, however, becomes increasingly important in the era of rising multimorbidity and several unfolding health crises [9–11].

In this study, we characterize profiles of disease histories in the USA and examine how their distribution in the population has changed before (until ∼2010) and during (from ∼2010) a period of stalling mortality. By shifting the focus from causes of death to disease histories, we gain a more comprehensive understanding of US population health to glean insights into how individual-level health trajectories aggregate to population-level mortality patterns.

The term “disease history” reflects the combination of diagnosed conditions that an individual has accumulated up to a given point in time, oriented on the clinical usage of “history of hypertension” or “history of diabetes,” referring to ever-diagnosed status rather than an observed temporal trajectory. These conditions often interact to shape long-term health outcomes and mortality risk [12, 13]. Disease histories can also enhance our understanding of the health dynamics behind deaths of despair—a key contributor to the US mortality disadvantage [14]. Such deaths are likely preceded by a history of mental health problems, chronic pain, or other conditions related to alcohol or drug use. Understanding how mortality trends are driven not only by changes in mortality risk, but also by changes in the proportion of the population living with those disease histories is often neglected in cause-of-death analyses.

In this study, we use information on self-reported health conditions (cancer, diabetes, CVD, mental health, internal diseases, obesity, and physical limitations) in the annual and nationally representative National Health Interview Survey (IPUMS-NHIS) (1997–2018) to (i) identify profiles of disease history in the US population; (ii) characterize these profiles by age, gender, and other sociodemographic factors; (iii) examine changes in the distribution of these profiles in the US population over age and time; and (iv) examine how changes in within-profile mortality risk and shifts in the population composition of disease-history profiles jointly shaped 5-year probabilities of death (measured from the interview date onward).

## Methods

### Data

We use data from the IPUMS-NHIS, which compiles and harmonizes NHIS data, to examine disease-history profiles in the years 1997–2018 (when health-condition survey items were asked consistently) [15]. The NHIS is a long-running annual sample of the non-institutionalized US population. Our analysis includes all individuals aged 30 to ≥85 years in the sample adult files with complete information on self-reported diseases and conditions used for the calculation of the disease histories (*N* = 510 372, 93.5% of the total sample from 1997–2018; see Supplementary Table 1). Records in the NHIS are linked to the US National Death Index through 31 December 2019. For the mortality analysis, we had to further restrict our sample to “sample adults” who were eligible for mortality follow-up (*N* = 493 818), which is a pre-specified subsample.

For estimations without considering mortality, we applied the annual NHIS person-weight (PERWEIGHT) to adjust for the complex survey design and achieve more representable results. The mortality analysis uses the mortality-specific person-weight (MORTWTSA), which is adjusted for sample adults who are ineligible for mortality follow-up.

### Measuring disease histories

Disease history was measured based on seven self-reported items about disease diagnoses and mental and physical problems, which, from a data-generating perspective, were deemed to be fully comparable over the observation period (see IPUMS-NHIS documentation) [15]. We included only conditions with a prevalence of ≥10% in the unadjusted sample for all NHIS waves combined. This was done to create more robust clusters and to minimize the risk of spurious findings arising from small disease groups. The selected diseases and health problems include cardiovascular diseases (hypertension, congestive heart failure, other heart diseases, angina pectoris, myocardial infarction, and stroke) captured as a count variable (0, 1, or 2+ CVD), as well as cancer (all types), diabetes, internal diseases (kidney problems, liver diseases, and ulcer), moderate-to-severe mental problems (measured by using the Kessler-6 scale with a threshold of 5 [16]), obesity defined as a body mass index (BMI) of ≥30, and the presence of physical limitations (see Table 1 in the appendix for further details), all captured in a dichotomous way (ever diagnosed/problem present: yes or no). CVD conditions are represented as a categorized count variable (0, 1, 2+) rather than six separate binary indicators, because equal weighting of indicator variables in latent class analysis (LCA) would otherwise risk solutions being driven primarily by CVD co-occurrence patterns rather than the broader cross-disease profiles that we aim to capture.

**Table 1:** Composition of disease-history groups by age, gender, race/ethnicity, education, and marital status, IPUMS-NHIS (1997–2018), ages ≥30 years.

Characteristic		Total	Disease-free	Cardiometabolic	Moderate Multimorbidity	Mental-physical burden	High-burden Multimorbidity
	Mean age (years)	53.78 (SD: 0.06)	49.12 (0.05)	56.81 (0.07)	68.95 (0.10)	55.93 (0.11)	64.80 (0.11)
Gender	Men	0.45	0.45	0.45	0.49	0.39	0.42
	Women	0.55	0.55	0.55	0.51	0.61	0.58
Race/ethnicity	Hispanic	0.1	0.1	0.1	0.06	0.09	0.1
	Non-Hispanic White	0.74	0.76	0.68	0.8	0.76	0.68
	Non-Hispanic Black	0.11	0.09	0.18	0.11	0.11	0.18
	Non-Hispanic Other/Multiple	0.04	0.05	0.03	0.04	0.03	0.04
Education	Less than high school	0.06	0.05	0.06	0.05	0.1	0.1
	High school diploma	0.37	0.35	0.41	0.38	0.45	0.48
	Some college	0.28	0.27	0.3	0.29	0.29	0.3
	Bachelor’s +	0.29	0.33	0.22	0.27	0.15	0.12
Marital status	Married	0.51	0.54	0.5	0.5	0.35	0.54
	Separated/divorced	0.19	0.17	0.18	0.21	0.28	0.17
	Widowed	0.14	0.13	0.14	0.13	0.16	0.13
	Living with partner	0.04	0.04	0.04	0.04	0.05	0.04
	Never married	0.12	0.12	0.14	0.12	0.16	0.12
5-year death probability	Men	0.062 (95% CI: 0.061–0.064)	0.037 (0.035–0.038)	0.051 (0.047–0.054)	0.096 (0.091–0.100)	0.144 (0.136–0.153)	0.178 (0.168–0.188)
	Women	0.043 (0.042–0.045)	0.024 (0.023–0.025)	0.036 (0.033–0.038)	0.074 (0.070–0.078)	0.095 (0.089–0.100)	0.126 (0.119–0.133)

Age-standardized using the age structure of the total weighted IPUMS-NHIS population in 2018 (most recent wave). Proportions sum to 1 within disease-history profiles.

### Statistical analyses

All analyses were stratified by gender (men/women). We used latent class analysis (LCA) to classify individuals with similar diseases and conditions into disease-history profiles. LCA is a statistical procedure that identifies a population’s homogeneous subgroups on the basis of the similarity of individual response patterns across a set of indicator variables, in this case disease diagnoses and mental and physical problems [17, 18]. We conducted the LCA separately for women and men, analysing all ages and years together. To determine the optimal number of classes, we employed the Elbow method with Akaike information criterion (AIC), Bayesian information criterion (BIC), and adjusted BIC (see supplemental materials for model fit statistics and validation).

After identifying the disease-history profiles, we characterized the mean age and age-standardized breakdown of educational attainment, race/ethnicity, and marital status for each profile. The age standardization is described in more detail in the Supplementary Materials. Next, we examined how the composition of disease-history profiles in the US population changed between 2000 and 2018 by plotting the cumulative age-specific proportion by disease-history type in the years 2000, 2009 and 2018.

We investigated the association between disease-history profiles and mortality over time. The mortality analysis was restricted to individuals aged 30–84 years; individuals aged ≥85 years were excluded due to the high institutionalized share at that age, which renders IPUMS-NHIS community-dwelling respondents an unrepresentatively healthy subgroup. Follow-up was limited to 5 years after interview, with the latest survey year being 2014, to ensure complete follow-up for all respondents. Individuals who did not die during the follow-up were censored at 5 years.

Logistic regression models, adjusted for the complex survey design, were fitted to estimate the 5-year probability of death as a function of disease-history profile, 5-year age group (30–34, …, 80–84 years), survey year in 3-year spans (1997–99, …, 2012–14), and their interaction. To ensure comparability across the periods, age-standardized estimated marginal means of the 5-year probability of death were computed by using the “emmeans” package [19]. We used the 1997–9 mortality-eligible age distribution as standard.

To quantify the contributions of compositional and within-profile mortality change to overall mortality change, we constructed a total mortality estimate as the sum, across disease-history profiles, of the product of the age-standardized prevalence and age-standardized 5-year probability of death for each profile. Two counterfactual scenarios were used to decompose the overall change: one isolating the contribution of shifting disease-history compositions and one isolating the contribution of improving within-profile mortality. Full details are provided in the Supplementary Materials. All analyses were performed in R (version 4.4.0) [20].

## Results

Figure 1 shows trends in the age-standardized prevalence of each condition used in the LCA. Obesity and diabetes show the largest increases, with obesity rising by almost 15 percentage points and diabetes by 6–8 percentage points over the period. Having at least one CVD condition was the most prevalent condition throughout, increasing from ∼40% to 45% before stabilizing. The prevalence of moderate and severe mental problems was relatively stable until 2010, particularly for women, after which it increased. Physical limitations and internal diseases declined slightly, while cancer prevalence increased modestly.

**Figure 1:**
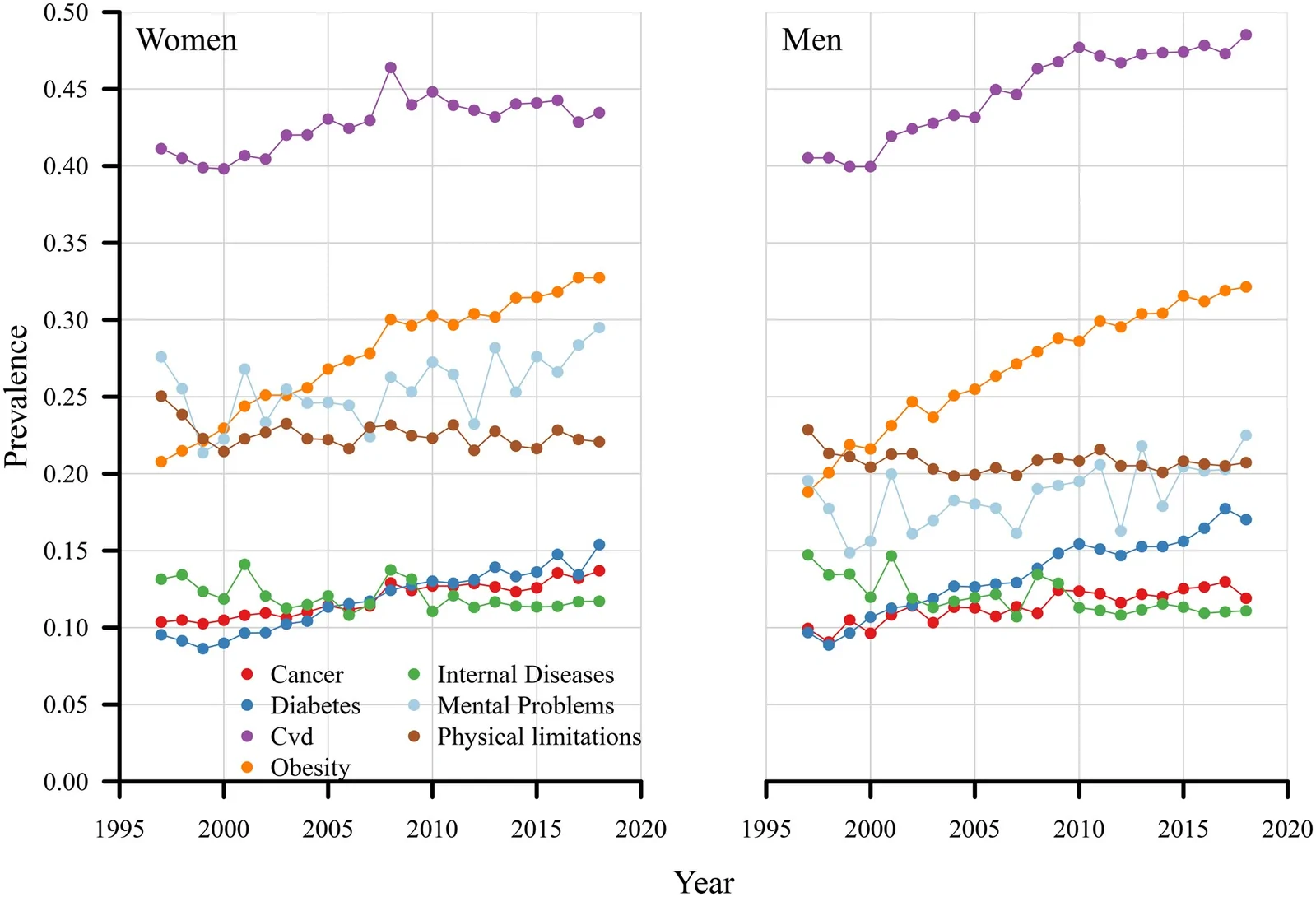
Age-standardized prevalence of health conditions used to construct disease-history profiles, ages ≥30 years, IPUMS-NHIS (1997–2018). Standardized to the age distribution in NHIS wave 2018 (total population); all estimates are weighted using the NHIS weights for sample adults (PERWEIGHT).

When these health conditions were used as indicator variables, the LCA fit statistics revealed that the diverse disease histories of respondents were best summarized by using five distinct classes, which were the same for men and for women (see Supplementary Figure 1). Figure 2 shows the five disease-history profiles and the probability of each condition within a profile. The figure shows the average across men and women.

**Figure 2:**
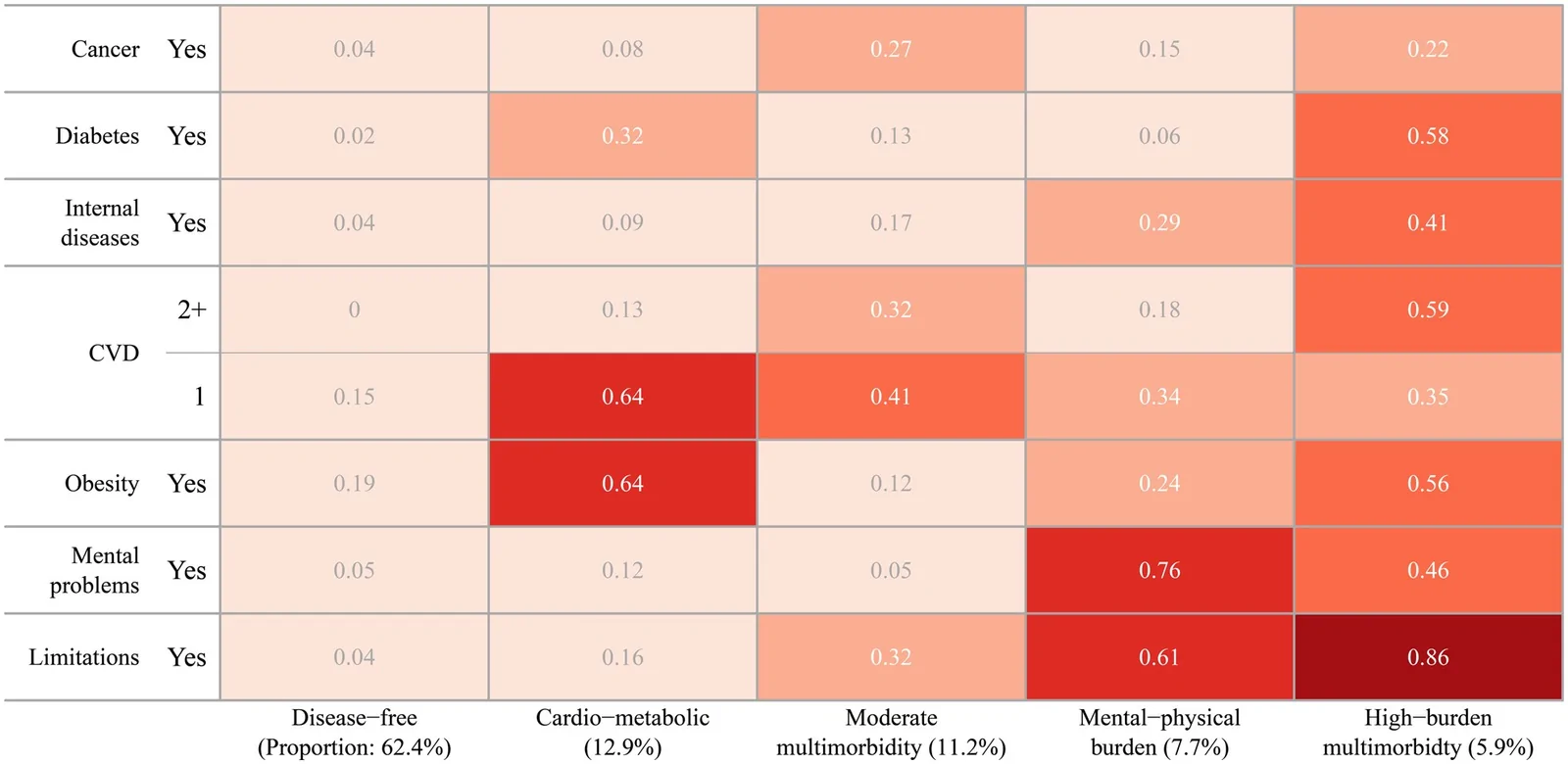
Disease-history profiles and corresponding probabilities of health conditions as average of women and men. IPUMS-NHIS (1997–2018), ages ≥30 years. Numbers are respective probabilities averaged across gender. Shading illustrates the magnitude of the probability, with darker fields indicating higher probabilities of reporting a disease.

The “disease-free” profile is characterized by low probabilities of all conditions. The “cardiometabolic” profile is defined by high probabilities of obesity and one CVD, moderate diabetes probability, and low probabilities of other conditions. Hypertension in particular forms the CVD burden in this profile (see Supplementary Table 4). The “moderate multimorbidity” profile has the highest probability of a previous cancer diagnosis, moderate probabilities of CVD and functional limitations, with one-third reporting physical limitations. The “mental–physical burden” profile is defined by particularly high probabilities of mental health problems and physical limitations, with low probabilities of other conditions. The “high-burden multimorbidity” profile shows elevated probabilities of almost all conditions, with 86% reporting functional limitations and elevated prevalence across all CVDs, including congestive heart failure (25.5%), myocardial infarction (21.1%), and stroke (18.5%).

Table 1 shows the population composition of each disease-history group. The “disease-free” group was the youngest (mean age 49 years) and the “moderate multimorbidity” group was the oldest (mean age 69 years). The “mental–physical burden” group was disproportionately female (61%). The “cardiometabolic” and “high-burden multimorbidity” groups had a higher proportion of non-Hispanic Black respondents (18% versus 11% in the total sample). Educational gradients were steep: the “high-burden multimorbidity” group had a lower share of bachelor’s degree holders compared with the “disease-free” group (12% versus 33%). The “mental–physical burden” group stood out for marital status, with the lowest proportion of married individuals (35% versus 51% total).

Figure 3 displays the age-group-specific proportions of each disease-history profile cumulatively for 2000, 2009, and 2018. Disease-profile composition changes strongly with age: the “disease-free” group declines sharply, while the “multimorbidity” groups increase. The “mental–physical burden” profile exhibits relatively stable proportions across the age groups, while the “cardiometabolic” profile reaches its largest share in midlife before declining thereafter. Figure 3 also reveals time trends in disease-history-profile compositions, visualized explicitly in Supplementary Figures 4 and 5. Over time, the “disease-free” group declines across all age groups (particularly between 2000 and 2009), replaced primarily by the “cardiometabolic” group, which shows the largest increases. The “high-burden multimorbidity” group shows a consistently increasing trend at ages ≥65 years. Patterns for the remaining profiles suggest broad stagnation over time.

**Figure 3:**
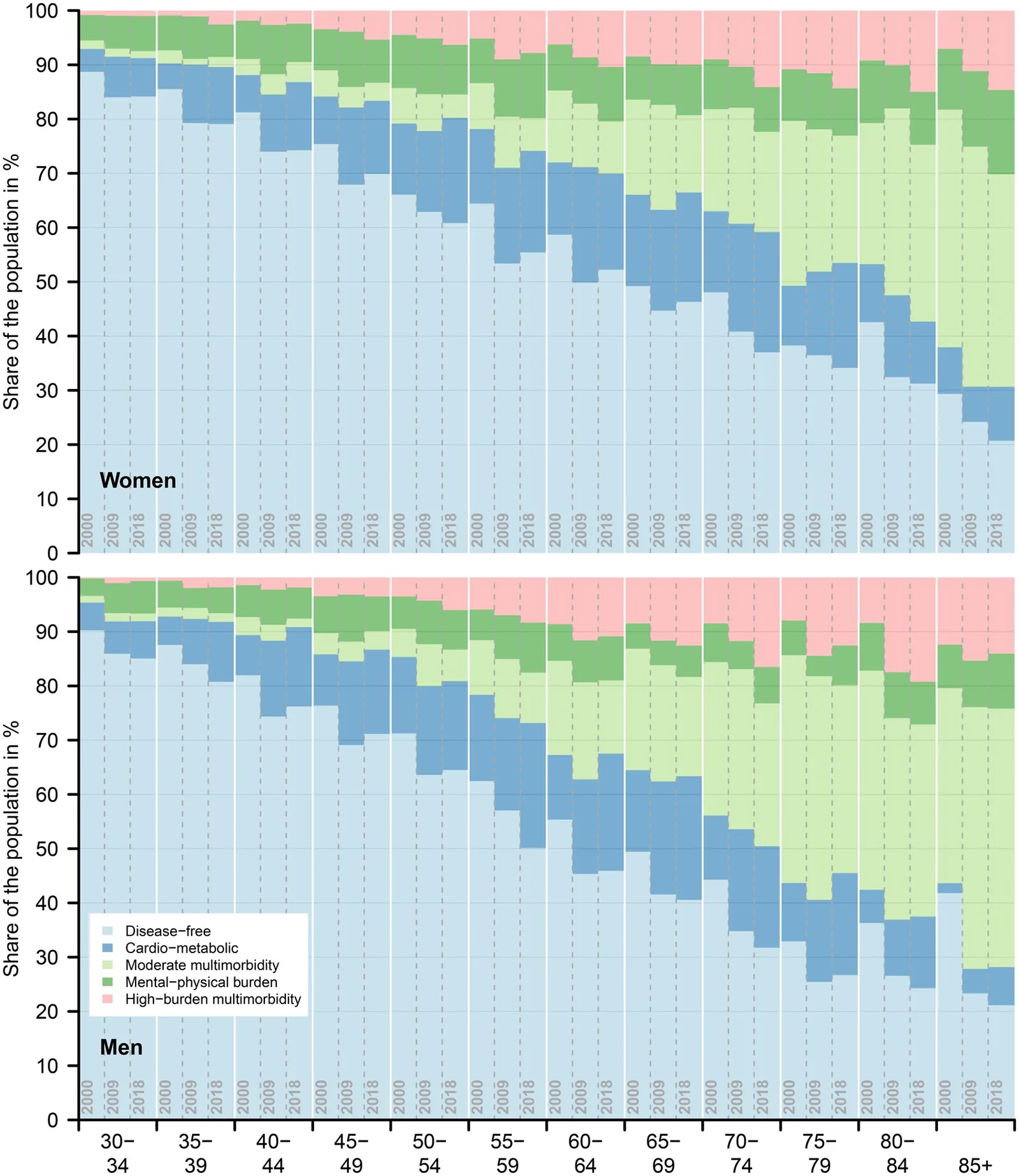
Age-group-specific cumulative proportions by disease-history profile in IPUMS-NHIS Waves 2000, 2009, and 2018, women and men. The composition is adjusted using the IPUMS-NHIS weights (PERWEIGHT).

Figure 4 shows the age-standardized 5-year probability of death following the NHIS interview by disease-history profile, separately by gender. For both women and men, the overall 5-year probability of death declined from 1997–1999 to the mid-2000s across most profiles. After this period, trends diverge and, given overlapping confidence intervals (CIs) between adjacent periods, should be interpreted with caution.

**Figure 4:**
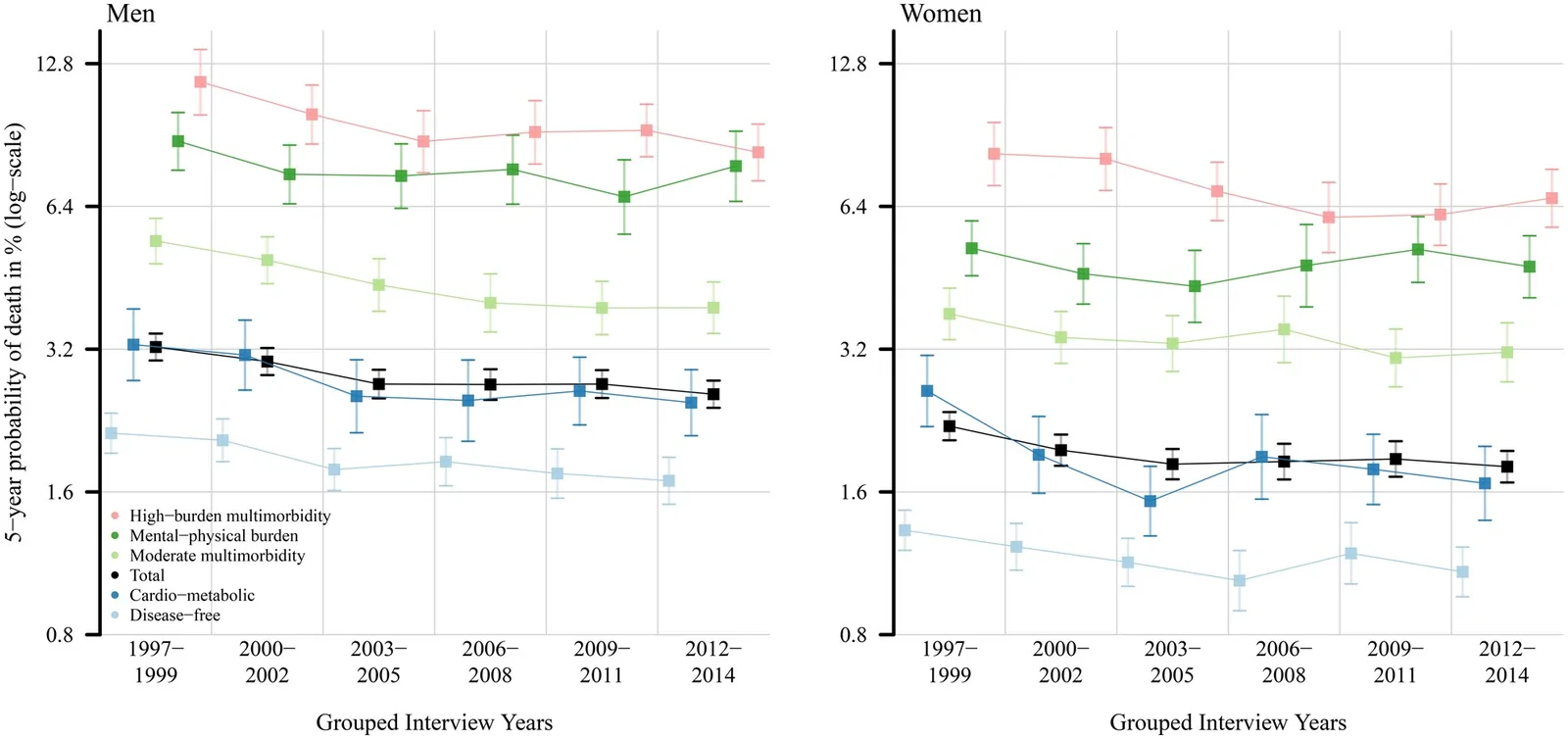
Five-year probability of death by disease-history type, IPUMS-NHIS (1997–2014, grouped into 3-year bins). The displayed probabilities are estimated marginal means that are age-standardized using the IPUMS-NHIS total population age structure of Waves 1997 to 1999 (combined). Probabilities of death were calculated using logistic regression models controlling for age and an interaction between year and disease-history type. Note that the calculation is restricted to the years 1997–2014, to allow sufficient follow-up time.

For the “disease-free” and “cardiometabolic” profiles, the CIs are sufficiently narrow and trends are sufficiently consistent across periods to support cautious interpretation of continued decline and relative stability, respectively. For the “high-burden multimorbidity” profile, a broadly increasing trend is visible in both genders from the mid-2000s onward, particularly among women. For the “moderate multimorbidity” and “mental–physical burden” profiles, the CIs are wider and adjacent periods overlap substantially, precluding strong conclusions about period-specific changes; the data are most consistent with broad stagnation in the mortality risk for these groups.

Figure 5 shows the observed 5-year probability of death and the two counterfactual scenarios for men and women, separately for all disease-history profiles combined and for the diseased population only. For both genders, the observed 5-year probability of death declined over the observation period (from 3.35% to 2.68% for men; from 2.27% to 1.86% for women). The counterfactual holding prevalences fixed at the 1997–1999 levels and changing only within-profile mortality shows a steeper decline than the observed levels. The counterfactual holding within-profile mortality fixed at the 1997–1999 levels and changing only the disease profile composition reveals a slight increase above the baseline level (+2.8% for men, +2.0% for women by 2012–14), confirming that compositional shifts partially offset mortality improvements over the period (see Supplementary Table 5). The observed decline therefore falls short of what within-profile mortality improvements alone would have produced, reflecting the attenuating effect of compositional change.

**Figure 5:**
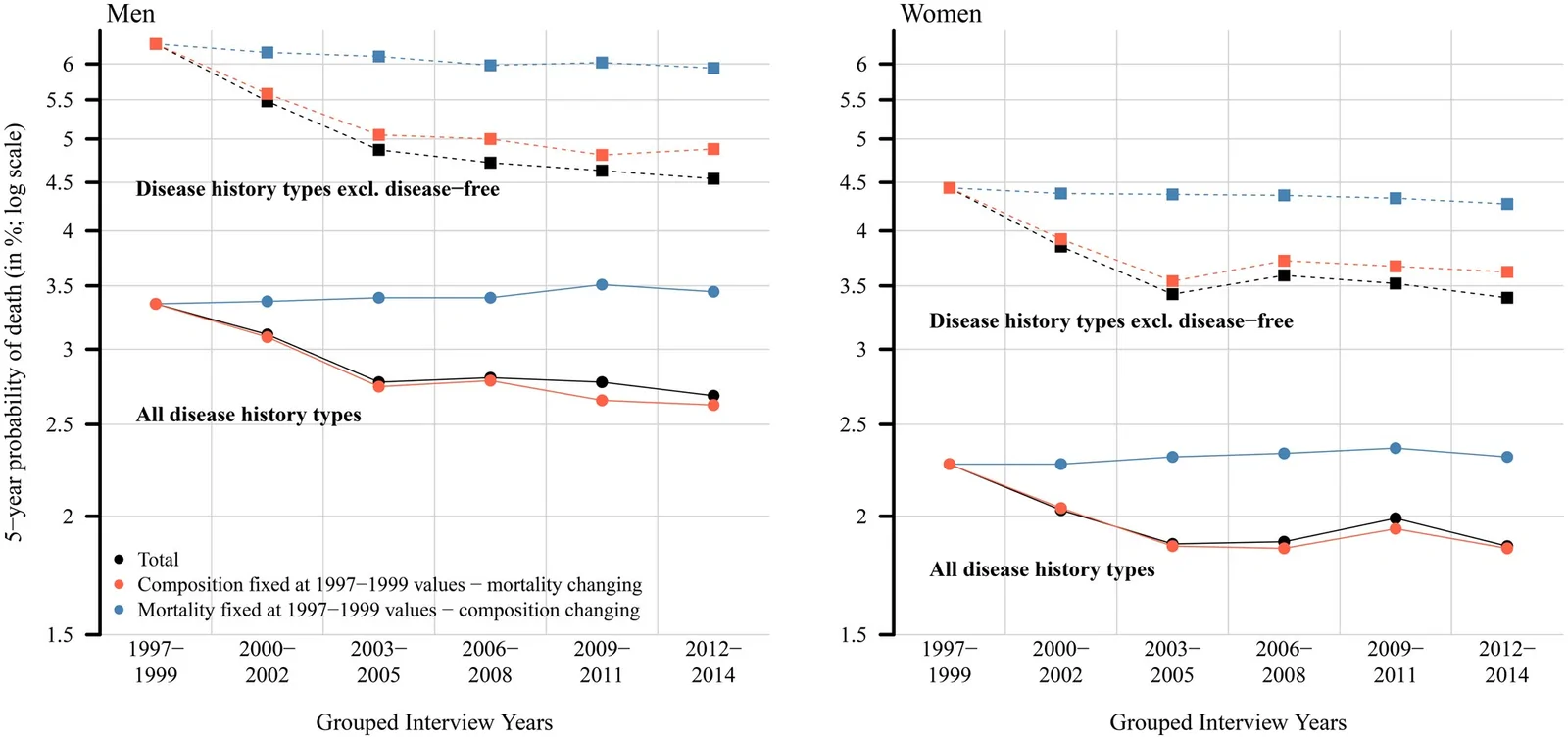
Counterfactual decomposition of changes in disease-history composition and within-type mortality in the total 5-year probability of death, by gender, IPUMS-NHIS (1997–2014, grouped into 3-year bins). Probabilities of death were calculated using logistic regression models controlling for age and an interaction between year groups and disease-history profiles. The age-standardized proportions are calculated using the IPUMS-NHIS total gender-specific populations combined for the Waves 2012 to 2014 as standard. Note that the calculation is restricted to the years 1997–2014, to allow sufficient follow-up time.

Among the diseased population only, the observed decline in the probability of death was steeper (from 6.30% to 4.54% for men; from 4.44% to 3.40% for women) and the composition counterfactual fell slightly below baseline (−5.8% for men, −3.9% for women), indicating mildly favorable redistribution among disease-history profiles within the diseased population. Within-profile mortality improvement was the dominant driver of the observed decline, but the favorable redistribution among disease-history profiles within the diseased population further amplified the decline beyond what mortality improvement alone would have produced, which is the opposite pattern to that observed in the full population (Supplementary Table 5).

## Discussion

This study traced trends in disease-history profiles in the USA, revealing that the disease-free portion of the population has decreased, while cardiometabolic and high multimorbidity profiles have become more common, especially at older ages. These trends align with evidence of rising obesity, cardiovascular disease, and diabetes, and reflect the broader increasing burden of multimorbidity shaped by cancer, internal diseases, and functional limitations [21–23]. The disease-history profile characterized by mental and physical burden exhibited increases among baby boomers (born in 1946–1964), consistently with evidence that this cohort has experienced the sharpest increases in alcohol-related deaths and suicides in the USA [24]. More broadly, successive US birth cohorts born since the mid-20th century are experiencing higher levels of multimorbidity and worse health trajectories than are earlier cohorts, with recent evidence suggesting that this pattern extends to cohorts born in the 1970s and beyond [25–28] and is not limited to the USA [29, 30].

An increased disease burden, however, is not in itself an indication of worsening population health. Rising prevalence may also reflect improved diagnostics or longer survival with disease, both of which should be associated with better outcomes, including lower mortality [31, 32]. We find mixed evidence with respect to trends in mortality risk. The 5-year mortality risk generally declined in the total sample and across disease-history profiles for those interviewed between 1997–1999 and 2003–2005 (translating to mortality observed in 1997–2010), but mortality flattened or increased thereafter for most disease-history profiles (in 2010–2019). Increasing mortality risk in the high-burden profiles is inconsistent with successful secondary or tertiary prevention. Moreover, stagnation in age-specific distributions of disease-history profiles since ∼2009 indicates that prevention efforts may have had only limited population-level effects.

National mortality trends are a function of both changes in disease burden and changes in the mortality risks associated with that burden. Our decomposition shows that mortality improvements “within” disease-history profiles are the overarching driver of declining mortality. Shifts in the population composition of disease histories, driven primarily by the shrinking disease-free share, exerted upward pressure on total population mortality, partially offsetting the within-group mortality improvements. Among the diseased population only, redistribution toward the milder cardiometabolic profile amplified the mortality decline beyond what within-profile improvement alone would have produced. However, this apparent compositional benefit among those with diseases should not be interpreted as a medical success: it reflects a growing prevalence of newly diseased individuals having disease earlier in life, typically through the cardiometabolic profile. The age-specific composition changes show that the cardiometabolic profile has grown at the expense of the disease-free profile across all age groups. The favorable redistribution among those with diseases is therefore itself a consequence of disease expansion rather than improved disease management. Taken together, these patterns confirm that shifts in disease-history composition have played a meaningful role in slowing US mortality improvements alongside changing within-profile mortality risks.

Examining disease-history profiles revealed the central importance of CVDs, which rarely operate in isolation, but typically co-occur with depression, diabetes, obesity, or other diseases [33, 34]. Four out of five profiles showed elevated CVD prevalence, with two exhibiting particularly high levels of multiple CVDs. These patterns suggest that the stagnation of US CVD mortality declines after 2010 [6] may have contributed to stalled mortality improvements across multiple disease-history profiles. Despite improved CVD management and advances in treating related conditions [13, 35], similar improvements have not translated into continued mortality declines in the USA, where increasing prevalence of additional risk factors and persistent disparities in care access continue to undermine progress. This is consistent with our finding that individuals with CVD-heavy profiles are more likely to be from lower socioeconomic backgrounds and racial-ethnic minorities, which are groups with a higher prevalence of obesity and more difficult access to healthcare [36–39]. Although the US slowdown is exceptionally strong, a similar pattern is emerging in several other high-income countries, raising concerns about the sustainability of long-term life-expectancy improvement [3, 14].

One limitation of this study is the difficulty in separating the ways in which disease histories shape mortality trends from the ways in which mortality trends shape changes in the composition of disease histories. Future research using longitudinal health data is needed to investigate this link in more depth and would also enable investigation of the sequence of disease diagnoses over time. Moreover, our analysis relied on self-reported ever-diagnoses. While this enabled coverage of a wide disease spectrum, more comprehensive and detailed disease information would have been beneficial. Mental health reporting may have been specifically affected by changes in social stigma and public awareness, potentially contributing to the observed increase in the “mental–physical burden” class independently of true prevalence changes. Additionally, BMI was derived from self-reported height and weight, which may have underestimated the obesity prevalence [40]. The absence of Alzheimer’s disease and related dementias is a further limitation: consistent dementia information is not available across the full observation period and the exclusion of the institutionalized population means that the NHIS captures only a selectively healthy picture of the dementia burden. Finally, pooling the LCA across all ages and years ensures consistent profiles for comparison over time, but does not capture potentially newly emerging profile types, representing a promising direction for future research.

The results from this study paint a richer picture of US population health than cause of death alone. All disease-history types except the “disease-free” group experienced either stagnation or increasing mortality from ∼2010 onward, suggesting that gains from medical progress in reducing mortality among the diseased have diminished over this period. The declining disease-free share and the growth in the cardiometabolic group as primary compositional drivers additionally counteracted partial improvements in within-profile mortality. Capturing changes to disease-history profiles can offer an opportunity to intervene before these shifting disease histories impact mortality.

## Ethics approval

The study used publicly available, de-identified NHIS data and did not require ethics approval.

## Supplementary Material

dyag217_Supplementary_Data

## Data Availability

All codes and other materials are accessible in the public repository at https://doi.org/10.17605/OSF.IO/YGK67. Researchers can access the IPUMS-NHIS data through the IPUMS Health Surveys portal (https://nhis.ipums.org/) by creating an account and submitting a data extract matching the specifications in this repository.
