# Supplementary material for: Sicker at all ages? Two decades of changing disease histories and mortality in the United States
Source: Int J Epidemiol. 2026 Oct 10;55(5):dyag217. doi: 10.1093/ije/dyag217 (PMC13654198; doi:10.1093/ije/dyag217)
Supplement: Supplementary file 1 [file dyag217_supplementary_data.pdf]

Supplemental materials: Sicker at All Ages? Two Decades of Changing Disease Histories and Mortality in the United States

Marcus Ebeling[1,2,3] Neil K Mehta [4] Leah Abrams [5] Mikko Myrskylä[1,3,6]

September 30, 2026

[1] Max Planck Institute for Demographic Research, Rostock, Germany
[2] Institute of Environmental Medicine, Karolinska Institutet, Stockholm, Sweden
[3] Max Planck – University of Helsinki Center for Social Inequalities in Population Health, Rostock, Germany
[4] Department of Epidemiology, University of Texas Medical Branch, Galveston (TX), USA
[5] Department of Community Health, Tufts University, Medford (MA), USA
[6] Helsinki Institute for Demography and Population Health, University of Helsinki, Helsinki, Finland

Age-Standardization of prevalence

Several prevalence calculations in the analysis are using age-standardization to control for the effect of varying age structures across disease history types and over time. In this section, we describe the applied standardization in more detail. The unstandardized prevalence, p, can be defined by

$$p = \frac{C}{N}$$

with C being the cases of interest (e.g. diseases or educational groups) and N being the respective underlying population (e.g. total population or all individuals assigned to certain disease history type). The equation can be rewritten and expressed as function of the age-structure of underlying population,

$$p = \sum_{x}^{\omega} \underbrace{\frac{N_x}{N}}_{c_x} \underbrace{\frac{C_x}{N_x}}_{p_x} = \sum_{x}^{\omega} c_x p_x$$

with N_x and C_x being the age-specific underlying population and cases of interest respectively. The age-specific prevalence is expressed by p_x and the share of the population at age x on the total underlying population is expressed by c_x. The latter needs to be replaced by a standard age-structure (e.g. the total population in a given year) , which we denote by c_x^{St}. Based on this formulation, the age-standardized total prevalence, p^{St}, can be expressed by

$$p^{St} = \sum_{x}^{\omega} c_x^{St} p_x.$$

Description of Indicator Variables for Latent Class Analysis

Table S1: Case numbers for each self-reported conditioned used in the Latent Class Analysis of Disease History Profiles, NHIS Waves 1997 to 2018 combined, women and men combined. Case numbers are unweighted.

Condition	Category	*N*
Ever told to have cancer (any type)	Yes	54,291
	No	491,107
	Missing or unknown	680
Ever told to have Diabetes	Yes/Borderline	66,091
	No	479,411
	Missing or unknown	576
CVD - Ever told to have coronary heart disease, heart condition/disease (other then previously mentioned), hypertension, angina pectoris, heart attack and stroke	Yes	221,388
	No	321,276
	Missing or unknown (in at least one)	3,414
Mental problems - Kessler-6-Scale	Score < 5	428,534
	Score ≥ 5	104,850
	Missing or unknown (in at least one of the six questions)	12,694
Physical limitation - Has any activity limitation	Limited in any way	115,264
	Not limited	430,160
	Missing or unknown	654
Obesity - Categorical body mass index	Obese with BMI ≥ 30	146,738
	BMI < 30	377,275
	Missing or unknown	22,065
Internal diseases - Ever told to have liver condition in the past 12 months, weak/failing kidneys in the past 12 months, and ulcer	Yes (reporting at least one)	63,024
	No	481,529
	Missing or unknown (in at least one)	1,525

Latent Class Analysis

Table S2: Posterior probabilities for assignment to the different disease-history groups, IPUMS NHIS Waves 1997 to 2018 are analyzed together but separately for women and men.

Sex	Assigned profile-Other profiles	1	2	3	4	5
Men	Disease-free (1)	0.838	0.028	0.064	0.068	0.001
	Cardio−metabolic diseases (2)	0.084	0.71	0.057	0.019	0.13
	Moderate multimorbidity (3)	0.151	0.027	0.671	0.097	0.055
	Mental & functional problems (4)	0.076	0.071	0.141	0.633	0.079
	High−burden multimorbidity (5)	0.002	0.088	0.105	0.098	0.707

Sex	Assigned profile-Other profiles	1	2	3	4	5
Women	Disease-free (1)	0.73	0.109	0.059	0.001	0.102
	Cardio−metabolic diseases (2)	0.132	0.709	0.003	0.07	0.086
	Moderate multimorbidity (3)	0.071	0.086	0.618	0.096	0.129
	Mental & functional problems (4)	0.001	0.04	0.068	0.825	0.066
	High−burden multimorbidity (5)	0.065	0.047	0.101	0.144	0.643

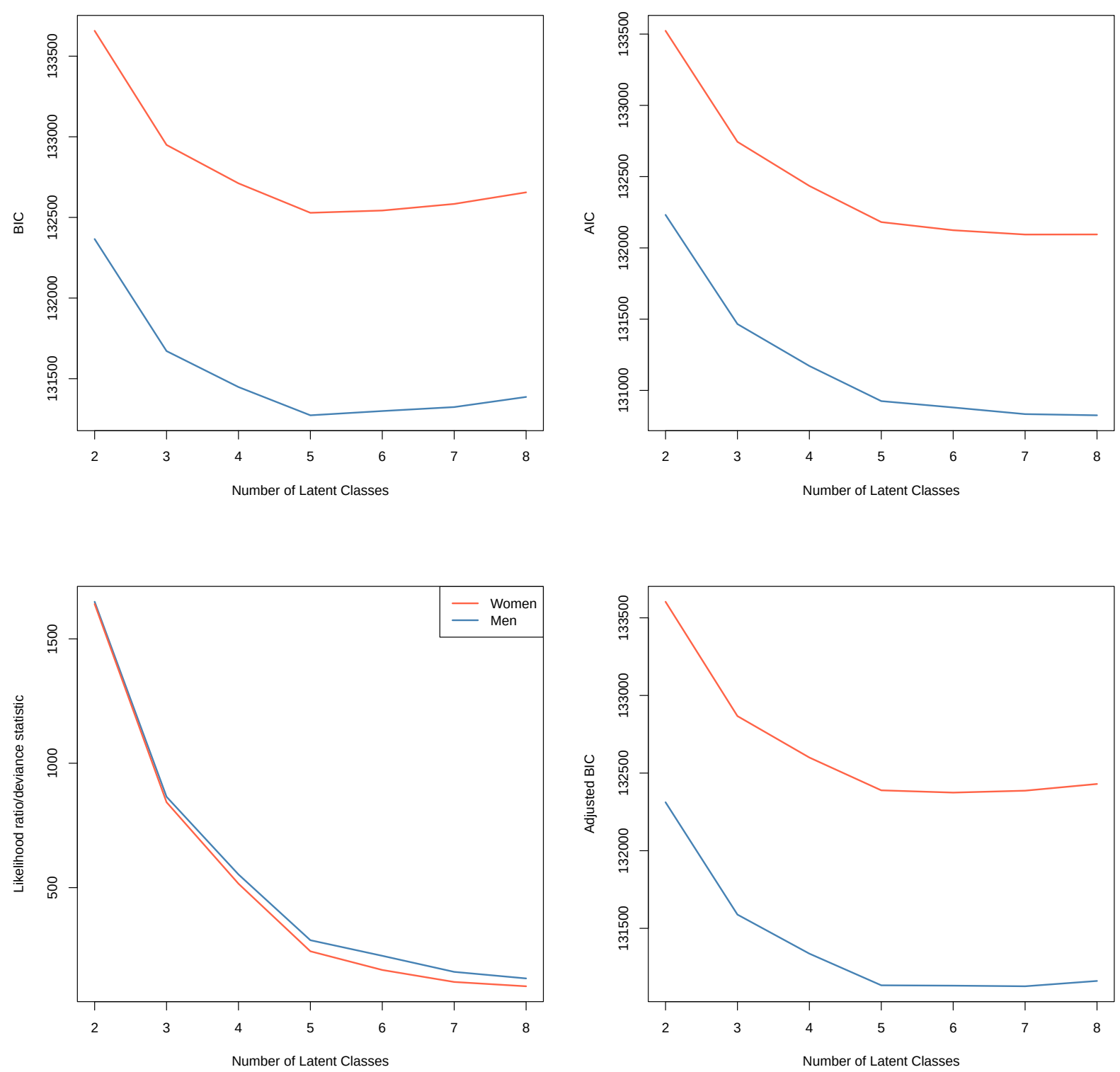

Figure S1: BIC, AIC, Adjusted BIC and Likelihood Ratio/Deviance Statistic based on a subsample of 20000 randomly selected respondents and LCA models with varying numbers of latent classes.

Table S3: Item-response probabilities by latent class profile using hypertension as separate indicator variable, IPUMS NHIS Waves 1997 to 2018 are analyzed together but separately for women and men. The previous model calibration also revealed the five-class model as the best solution.

	**Women**				
**Indicator**	**Class A** (64.73%)	**Class B** (13.38%)	**Class C** (9.87%)	**Class D** (5.76%)	**Class E** (6.25%)
Cancer (Yes)	0.03	0.08	0.31	0.10	0.22
Diabetes (Yes)	0.02	0.33	0.15	0.08	0.55
CVD 0	0.97	0.79	0.46	0.82	0.30
CVD 1	0.03	0.12	0.30	0.12	0.29
CVD 2+	0.00	0.09	0.24	0.06	0.41
Mental problems (Yes)	0.10	0.10	0.06	0.72	0.49
Limitations (Yes)	0.04	0.14	0.31	0.67	0.85
Obesity (Yes)	0.19	0.64	0.08	0.21	0.50
Hypertension (Yes)	0.13	0.76	0.60	0.34	0.91
Internal diseases (Yes)	0.04	0.09	0.18	0.29	0.40
	**Men**				
**Indicator**	**Class A** (65.05%)	**Class B** (13.66%)	**Class C** (10.13%)	**Class D** (5.85%)	**Class E** (5.30%)
Cancer (Yes)	0.03	0.09	0.28	0.14	0.25
Diabetes (Yes)	0.02	0.34	0.16	0.08	0.56
CVD 0	0.98	0.77	0.51	0.74	0.35
CVD 1	0.02	0.12	0.28	0.18	0.25
CVD 2+	0.00	0.11	0.20	0.07	0.40
Mental problems (Yes)	0.09	0.11	0.02	0.79	0.44
Limitations (Yes)	0.04	0.14	0.30	0.65	0.86
Obesity (Yes)	0.19	0.63	0.07	0.24	0.51
Hypertension (Yes)	0.12	0.77	0.58	0.43	0.87
Internal diseases (Yes)	0.05	0.08	0.17	0.27	0.46

Additional Results

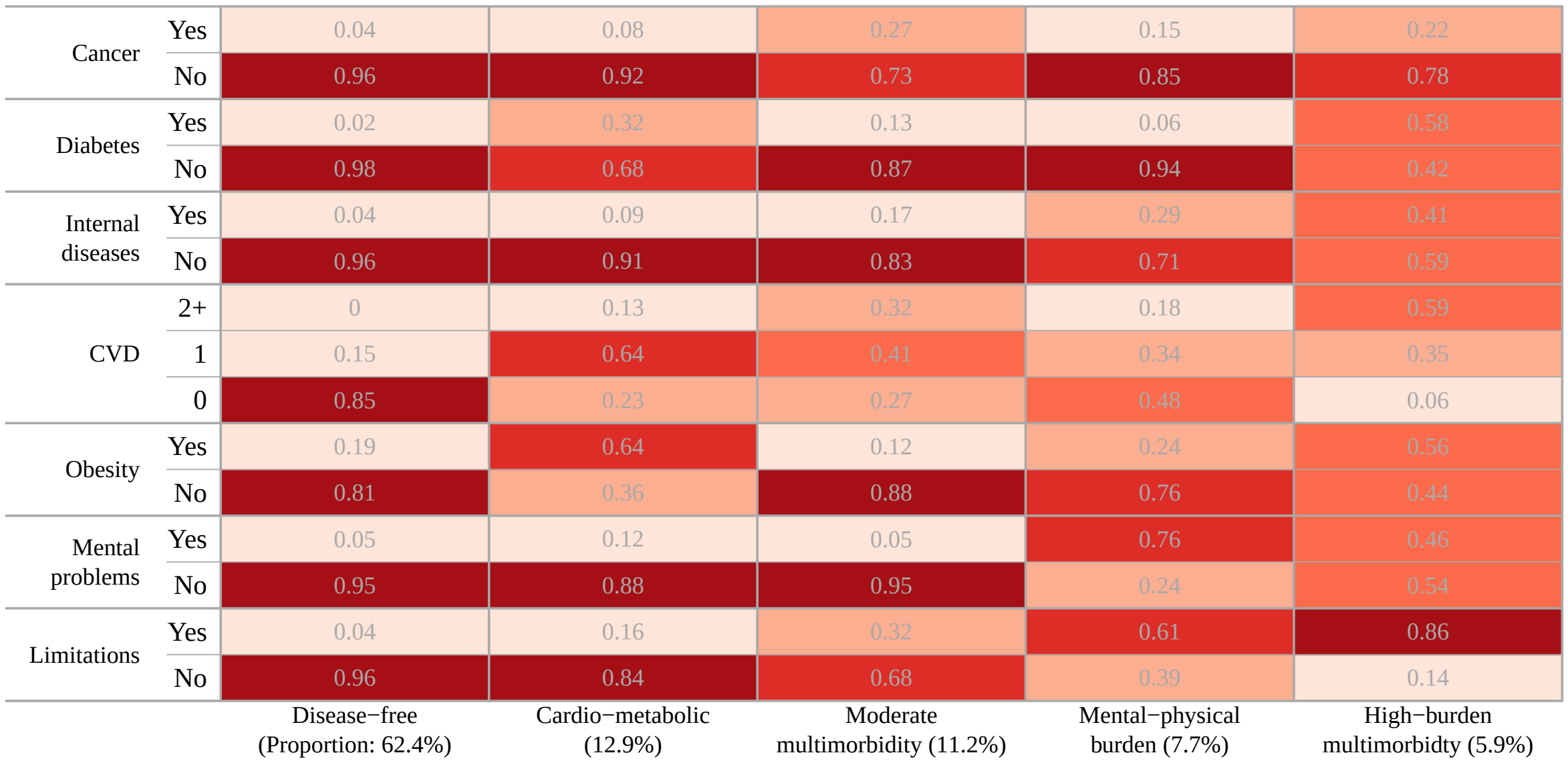

Figure S2: Disease history profiles and corresponding probabilities of health conditions. IPUMS-NHIS (1997-2018), ages 30+. Numbers are respective probabilities averaged across gender.

Table S4: Age-standardized prevalence of individual cardiovascular conditions by disease history type, IPUMS-NHIS 1997–2018

	**Cardiovascular condition (%)**					
**Disease history type**	Angina pectoris	Congestive heart failure	Other heart diseases	Hypertension	Myocardial infarction	Stroke
Disease-free	0.2	0.3	2.2	16.6	0.3	0.3
Cardio-metabolic	2.3	4.7	9.9	81.3	3.5	2.2
Moderate multimorbidity	8.7	16.5	30.3	66.2	12.3	9.4
Mental-physical burden	5.9	8.6	15.6	44.1	7.3	7.2
High-burden multimorbidity	15.5	25.5	36.4	83.7	21.1	18.5

Notes: Prevalences are age-standardized to the 2018 IPUMS-NHIS population. All values expressed as percentages. NHIS waves 1997–2018 combined.

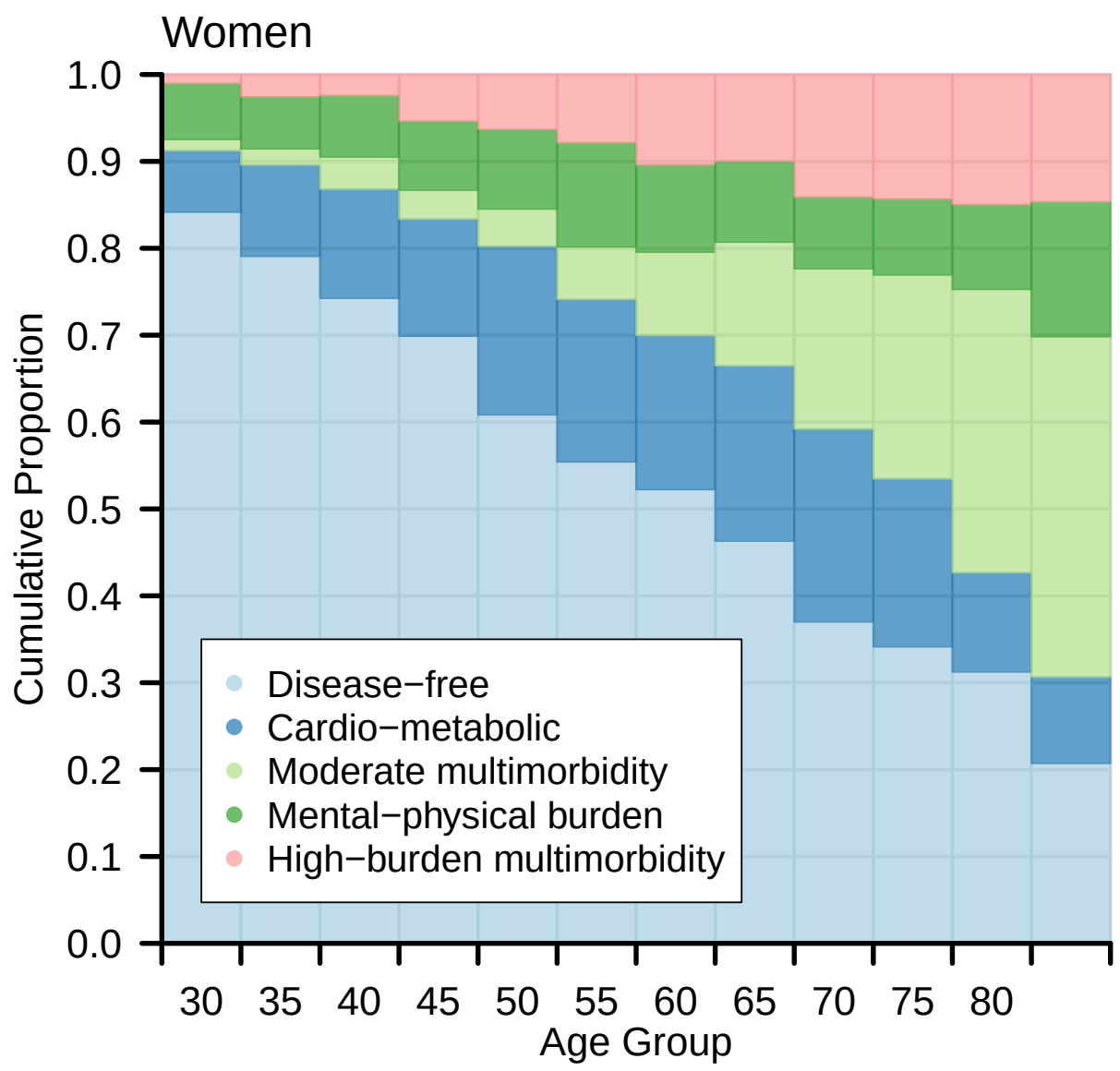

Figure S3: Population composition by disease history profile and age group. IPUMS-NHIS 2018, ages 30+. The composition is adjusted using the IPUMS NHIS weights (PERWEIGHT)

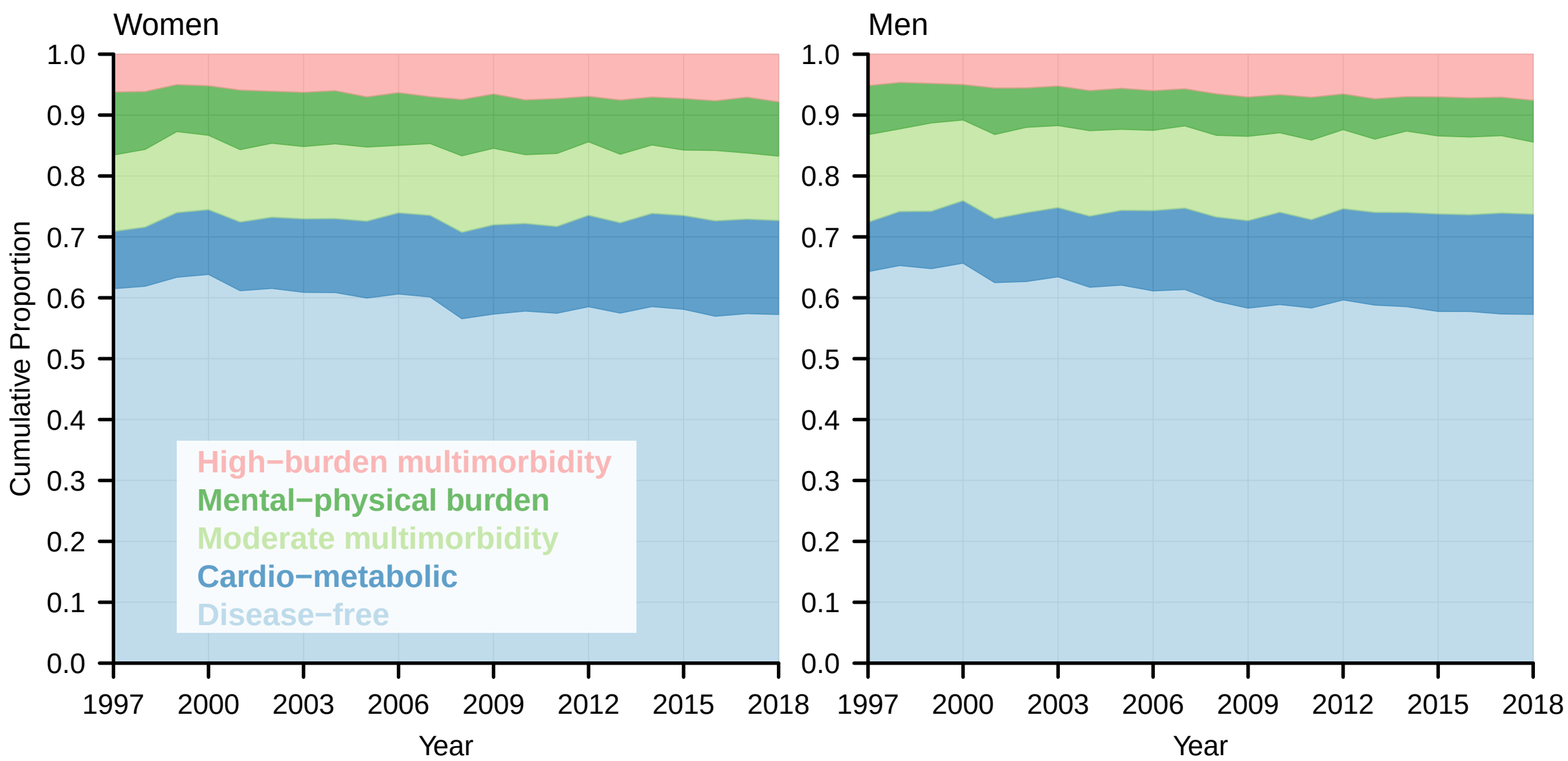

Figure S4: Change over time (1997-2018) in total population composition of disease history groups and age-specific composition in NHIS Wave 2018, IPUMS-NHIS (ages 30+). The composition is adjusted using the IPUMS NHIS weights (PERWEIGHT)

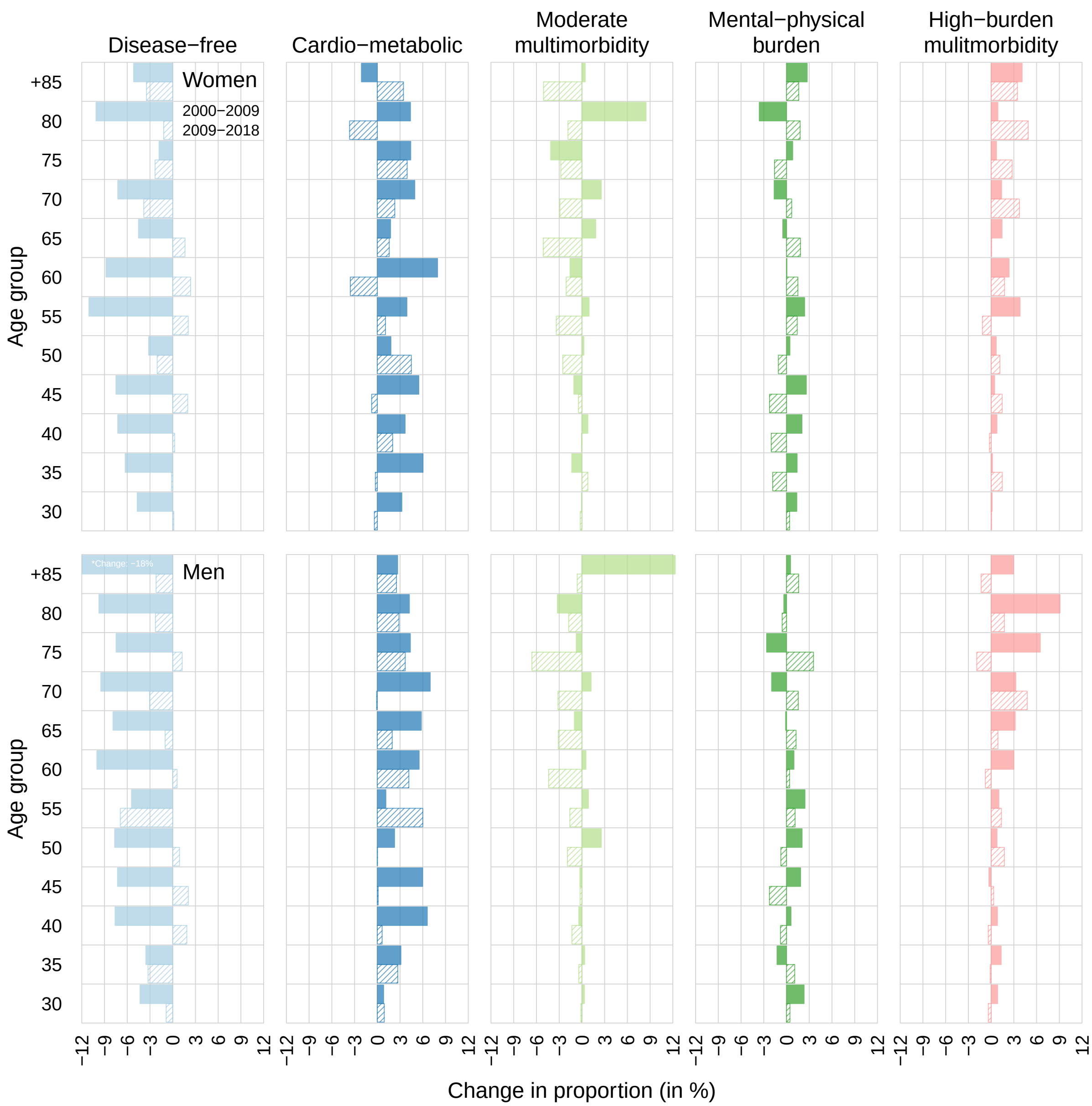

Figure S5: Age-specific change in the proportions in each disease history group in 2000-2009 and in 2009-2018, IPUMS NHIS. The composition is adjusted using the IPUMS NHIS weights (PERWEIGHT)

Table S5: Counterfactual decomposition of the five-year probability of death, IPUMS-NHIS 1997–2014

	**All disease history types**						**Diseased only (excl. disease-free, renormalized)**					
	5-year prob. of death			% change from baseline			5-year prob. of death			% change from baseline		
	Observed	Mort. fixed	Prev. fixed	Observed	Mort. fixed	Prev. fixed	Observed	Mort. fixed	Prev. fixed	Observed	Mort. fixed	Prev. fixed
Men												
1997–1999	3.35	3.35	3.35	0.0	0.0	0.0	6.30	6.30	6.30	0.0	0.0	0.0
2000–2002	3.11	3.37	3.09	−7.3	0.6	−7.8	5.48	6.17	5.58	−13.0	−2.0	−11.4
2003–2005	2.77	3.40	2.74	−17.5	1.3	−18.3	4.87	6.11	5.05	−22.7	−3.0	−19.9
2006–2008	2.80	3.40	2.78	−16.5	1.4	−17.2	4.72	5.98	5.00	−25.1	−5.2	−20.7
2009–2011	2.77	3.51	2.65	−17.4	4.6	−21.1	4.63	6.02	4.81	−26.5	−4.4	−23.8
2012–2014	2.68	3.45	2.62	−20.1	2.8	−21.7	4.54	5.94	4.88	−27.9	−5.8	−22.6
Women												
1997–1999	2.27	2.27	2.27	0.0	0.0	0.0	4.44	4.44	4.44	0.0	0.0	0.0
2000–2002	2.03	2.27	2.04	−10.3	0.0	−10.1	3.85	4.38	3.92	−13.3	−1.3	−11.7
2003–2005	1.87	2.31	1.86	−17.3	1.7	−18.0	3.43	4.37	3.54	−22.8	−1.7	−20.4
2006–2008	1.88	2.33	1.85	−17.1	2.7	−18.5	3.59	4.36	3.72	−19.3	−1.8	−16.2
2009–2011	1.99	2.36	1.94	−12.3	4.1	−14.6	3.52	4.33	3.67	−20.8	−2.5	−17.4
2012–2014	1.86	2.31	1.85	−18.0	2.0	−18.4	3.40	4.27	3.62	−23.5	−3.9	−18.5

Notes: Observed = $B(t) = \sum_k p_k(t) \times q_k(t)$. Mort. fixed = $B_p(t) = \sum_k p_k(t) \times q_k(1997\text{–}1999)$, holds within-profile mortality fixed at the 1997–1999 baseline (isolates the composition effect). Prev. fixed = $B_q(t) = \sum_k p_k(1997\text{–}1999) \times q_k(t)$, holds prevalences fixed at baseline (isolates the mortality improvement effect). Diseased only restricts to disease history types 2–5 with prevalences renormalized within the diseased population. Five-year probabilities expressed as percentages. All estimates standardized to the 1997–1999 IPUMS-NHIS mortality-eligible age distribution.
